# 

**Published:** 1896-07

**Authors:** 


### *INDEX NUMBER.*

### CONTENTS FOR JULY, 1896.

ORIGINAL COMMUNICATIONS. PAGE.

The Evolution of the Surgical Treatment of the Broad Ligament Pedicle. By LAWSON TAIT, F. R. C. S ..... 929

The Relations of Medical Examining Boards to the State, to the Schools and to Each Other. By WILLIAM WARREN POTTER, M. D ..... 938

Historical Sketch of the Work of the Board of Censors of the Medical Society of the County of Erie. By EDWARD STORCK, M. D ..... 951

CLINICAL LECTURE.

Removal of Gall Stones—Ovarian Cyst—Spleen—Kidney and Ureter, en Masse ..... 954

SOCIETY PROCEEDINGS.

Proceedings of the Sixth Annual Meeting of the National Confederation of State Medical Examining and Licensing Boards, held at Atlanta, Ga., May 4, 1896 ..... 958

Medical Society of the County of Erie ..... 965

Army Medical Examinations ..... 968

EDITORIAL.

The May Meetings at Atlanta ..... 970

The Medical Department of Niagara University ..... 973

Topics of the Month ..... 974

Personal ..... 974

Obituary ..... 975

Society Meetings ..... 975

Medical College Notes ..... 977

Reviews ..... 978

